# Investigating the Association between Serum and Hematological Biomarkers and Neonatal Sepsis in Newborns with Premature Rupture of Membranes: A Retrospective Study

**DOI:** 10.3390/children11010124

**Published:** 2024-01-18

**Authors:** Maura-Adelina Hincu, Gabriela-Ildiko Zonda, Petronela Vicoveanu, Valeriu Harabor, Anamaria Harabor, Alexandru Carauleanu, Alina-Sînziana Melinte-Popescu, Marian Melinte-Popescu, Elena Mihalceanu, Mariana Stuparu-Cretu, Ingrid-Andrada Vasilache, Dragos Nemescu, Luminita Paduraru

**Affiliations:** 1 Division of Neonatology, Department of Mother and Child Care, “Grigore T. Popa” University of Medicine and Pharmacy, 700115 Iasi, Romaniaale.carauleanu@umfiasi.ro (A.C.); dragos.nemescu@umfiasi.ro (D.N.);; 2 Department of Mother and Child Care, “Grigore T. Popa” University of Medicine and Pharmacy, 700115 Iasi, Romania; petronela.pintilie@umfiasi.ro; 3 Clinical and Surgical Department, Faculty of Medicine and Pharmacy, ‘Dunarea de Jos’ University, 800216 Galati, Romania; valeriu.harabor@ugal.ro (V.H.); ana.harabor@ugal.ro (A.H.); mariana.stuparu@ugal.ro (M.S.-C.);; 4 Department of Mother and Newborn Care, Faculty of Medicine and Biological Sciences, ‘Ștefan cel Mare’ University, 720229 Suceava, Romania; 5 Department of Internal Medicine, Faculty of Medicine and Biological Sciences, ‘Ștefan cel Mare’ University, 720229 Suceava, Romania

**Keywords:** early-onset sepsis, biomarker, c-reactive protein, procalcitonin, white blood count, antibiotics

## Abstract

(1) Background: Neonatal early-onset sepsis (EOS) is associated with important mortality and morbidity. The aims of this study were to evaluate the association between serum and hematological biomarkers with early onset neonatal sepsis in a cohort of patients with prolonged rupture of membranes (PROM) and to calculate their diagnostic accuracy. (2) Methods: A retrospective cohort study was conducted on 1355 newborns with PROM admitted between January 2017 and March 2020, who were divided into two groups: group A, with PROM ≥ 18 h, and group B, with ROM < 18 h. Both groups were further split into subgroups: proven sepsis, presumed sepsis, and no sepsis. Descriptive statistics, analysis of variance (ANOVA) and a Random Effects Generalized Least Squares (GLS) regression were used to evaluate the data. (3) Results: The statistically significant predictors of neonatal sepsis were the high white blood cell count from the first (*p* = 0.005) and third day (*p* = 0.028), and high C-reactive protein (CRP) values from the first day (*p* = 0.004). Procalcitonin (area under the curve—AUC = 0.78) and CRP (AUC = 0.76) measured on the first day had the best predictive performance for early-onset neonatal sepsis. (4) Conclusions: Our results outline the feasibility of using procalcitonin and CRP measured on the first day taken individually in order to increase the detection rate of early-onset neonatal sepsis, in the absence of positive blood culture.

## 1. Introduction

Prolonged rupture of membranes (PROM) occurs in 8–10% of pregnancies [1,2] and may be complicated by the microbial invasion of the amniotic cavity, inducing histological chorioamnionitis, intraamniotic inflammation, premature birth, and neonatal infection [3]. Approximately one-third of spontaneous preterm births are associated with premature prolonged rupture of membranes (PPROM) [4]. Other causes of preterm birth include multiple pregnancies, congenital abnormalities, chronic maternal conditions (diabetes, hypertension, autoimmune disorders, abdominal or uterine tumors, etc.), in vitro fertilization (IVF), extremes of ages or weight, maternal use of illicit drugs or alcohol, psychiatric comorbidities, or current pregnancy complications that require an iatrogenic preterm birth [5,6,7,8,9,10,11,12,13,14,15]. Previous studies have proven that the lower the gestational age (GA) at which the membranes rupture, the greater the probability of infection [16,17].

Obstetricians need to choose between conservative management, which could lead to chorioamnionitis and neonatal early-onset sepsis (EOS), and premature delivery, which is associated with prematurity complications. EOS was defined as an infection due to organisms acquired before and during delivery, occurring in the first 72 h of life [18]. However, there are studies that extend the definition of EOS determined by *group B Streptococcus* (GBS) up to 7 days of life [19,20,21].

EOS is a challenge for neonatologists, being an invasive infection often suspected, but rarely diagnosed, with a proven diagnosis of 0.8 in 1000 births [22]. Due to significant mortality and severe complications associated with neonatal sepsis, early initiation of broad-spectrum antibiotics is the first step to decrease morbidity and mortality [23,24]. Positive blood culture is the only certain diagnostic tool, but its results are confirmed within a 36–48 h time frame. Even in the presence of specific signs and symptoms, less than 1% of newborns with suspected sepsis have a positive blood culture [25].

In order to aid in the prompt detection and precise diagnosis of neonatal sepsis, more modern molecular approaches and nonculture-based techniques are required. Although the white blood cells (WBC), the immature/total neutrophils ratio (I/T), and the number of platelets (PLT) do not show high sensitivity and specificity to diagnose infections, these markers are the most used in neonatal units [26].

Leukopenia (WBC count 5000/mm^3^) has a high specificity (91%), but a low sensitivity (29%) for the diagnosis of newborn sepsis according to a literature review by Sharma et al. [27]. I/T ratio may be the most accurate predictor of neonatal sepsis when compared to other hematological indicators, with a value greater than 0.27 in term newborns, and greater than 0.22 in preterm newborns being indicative of neonatal sepsis, but the serum level of this biomarker fluctuates with gestational age and postnatal age [28].

It has been shown that serial C-reactive protein (CRP) measurements increase its sensitivity and negative predictive value for neonatal sepsis, and may be beneficial for assessing the treatment response of affected neonates under antibiotic therapy [29]. Procalcitonin (PCT) could also be considered a promising biomarker for neonatal sepsis due to high sensitivity (81%; 95% CI: 74–87%) and specificity (79%; 95% CI: 69–87%) values, as reported in a meta-analysis [30].

Other literature data indicated that currently determined markers in the newborns’ serum may be elevated due to other factors unrelated to infection, such as hypertension or maternal fever, prolonged labor, perinatal asphyxia, meconium aspiration syndrome, respiratory distress syndrome, intracranial hemorrhage, or pneumothorax [31,32].

The aims of this study were to evaluate the association between specific serum and hematological biomarkers with early onset neonatal sepsis in a cohort of patients with PROM and to calculate their diagnostic accuracy.

## 2. Materials and Methods

### 2.1. Study Design

An observational retrospective cohort study was conducted using the database of patients admitted to a level III neonatal center of “Cuza Voda” Clinical Hospital of Obstetrics and Gynecology, between January 2017 and March 2020. The medical charts of the neonates were retrospectively reviewed. Information on the gestational age, weight, gender, mode of delivery, need for resuscitation, Apgar score, risk factors for infection, and clinical signs of sepsis were extracted.

Access to patient’s medical records and the study protocol was approved by the Institutional Ethics Committee of the regional hospital (No. 5332/21 May 2020). Newborns’ personal data were anonymized prior to analysis.

### 2.2. Definitions and Study Population

A total of 1355 medical records of neonates were analyzed. Newborns with PROM and postnatal age <24 h were included in the study. The gestational age (GA) ranged from 23 to 43 weeks. Exclusion criteria were infants born at less than 23 weeks of gestation, infants with congenital anomalies, and the absence/incomplete sepsis screening according to the unit protocol.

The population was divided into two groups. Group A (n = 826 patients) included neonates with prolonged rupture of membranes, longer than 18 h before birth, while the infants with ROM (rupture of membranes) less than 18 h were assigned to group B (n = 529 patients). The cut-off of 18 h was chosen in accordance with our local protocols.

The diagnosis of sepsis was performed according to the criteria proposed by the European Medicines Agency (EMA) [33]. For the secondary analysis, we further stratified the neonates into three subgroups: proven EOS (subgroups A1 and B1), presumed EOS (subgroups A2 and B2), and absence of EOS–control group (subgroups A3 and B3). Assignment of patients in one of the subgroups was made according to our local institutional criteria and laboratory ranges:

- No sepsis (absence of clinical signs suggestive for sepsis; CRP < 6 mg/L; PCT < 0.5 ng/mL; hematological and biochemical parameters within normal limits; and negative blood culture);
- Presumed EOS ≥3 clinical signs suggestive of sepsis; CRP ≥ 10 mg/L; PCT > 0.5 ng/mL; ≥2 altered serum parameters, other than CRP or PCT: WBC, I/T, PLT; and negative blood culture;
- Proven EOS ≥ 3 clinical signs suggestive of sepsis; CRP ≥ 10 mg/L; PCT > 0.5 ng/mL; ≥2 altered serum parameters, other than CRP or PCT; and positive blood culture.

The suspicion of infection was assessed on admission in all newborns with clinical signs suggesting infection including respiratory signs: apnea, tachypnea, retractions, need for supplemental oxygen/respiratory support; cardio-circulatory signs: tachycardia/bradycardia, hypotension, or impaired peripheral perfusion (mottled skin, cold extremities); oliguria (urine output < 1 mL/kg/h); temperature instability, hypothermia or hyperthermia; gastrointestinal signs: vomiting, abdominal distension, bilious/bloody gastric aspirates; skin and subcutaneous signs: petechial rash or scleroderma; and neurological signs: irritability, lethargy, hypotonia, weak sucking.

The sepsis panel included a complete blood count, C-reactive protein, procalcitonin, fibrinogen, and blood culture, which was performed at admission, at 72 h of life, and on the 5th day of life, except for the blood culture and procalcitonin levels that were determined on a single occasion, at admission. Serum and hematological parameters were flagged as abnormal as follows: leukopenia (<4 × 10^9^ cells/L) or leukocytosis (>20 × 10^9^ cells/L), immature to total neutrophils ratio (I/T) > 0.2, thrombocytopenia (<100 × 10^9^ cells/L) and inflammatory syndrome (CRP > 10 mg/L or PCT ≥ 10 ng/mL). According to the local protocol, antibiotic therapy was started in all neonates with clinical suspicion of infection and/or risk factors for sepsis (including rupture of membranes).

An amniotic fluid culture was performed for all pregnant patients with ruptured membranes before delivery. Clinical chorioamnionitis was diagnosed in the presence of maternal fever with two of the following: maternal tachycardia, fetal tachycardia, uterine tenderness, foul odor of amniotic fluid, or maternal leukocytosis [34]. Leukocytosis (>10.000/mm^3^) and high CRP values (>6 mg/L) were considered signs of maternal inflammatory syndrome according to the local laboratory thresholds. Maternal fever was considered when the core body temperature was higher than 38 °C.

### 2.3. Statistical Analysis

The paired sample *t*-test and independent-sample *t*-test were used for continuous variables. Continuous variables were presented as the mean +/− standard deviation (SD) or median and interquartile ranges. Categorical variables were presented as frequencies with corresponding percentages.

Analysis of variance (ANOVA) with the Bonferroni post-hoc test was used to determine whether or not there is a statistically significant difference between the means of serum and hematological biomarkers (WBC, CRP, and fibrinogen) between subgroups, and boxplots were used for graphical representations of these differences.

A Random Effects Generalized Least Squares (GLS) regression was used to measure the association between predictor variables, such as serum and hematological biomarkers levels (WBC, CRP, and fibrinogen) measured on three different occasions, and an outcome variable, presumed early-onset neonatal sepsis. The performance of laboratory biomarkers in the diagnosis of EOS was calculated by using the area under the curve-receiver operating curve (AUC). These analyses were performed using STATA SE (version 15, StataCorp LLC, College Station, TX, USA). A *p*-value of less than 0.05 was considered statistically significant.

## 3. Results

**Figure 1:**
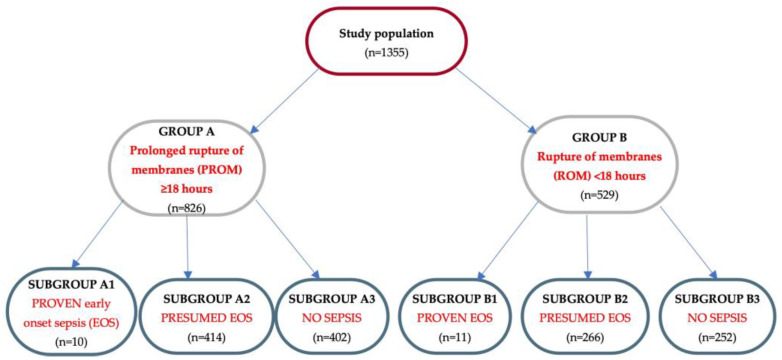
Flowchart of the study group distribution. Legend: EOS—early onset sepsis; PROM—prolonged rupture of membranes; ROM—rupture of membranes.

According to the criteria mentioned, 826 newborns were assigned to the group with PROM ≥ 18 h, out of which 10 were included in the proven EOS group (0.7%), 414 neonates in the probable EOS category (30.6%), and 402 in the control group, without sepsis (29.7%). Another 549 neonates with PROM < 18 h were segregated into the proven EOS category (n = 11 patients, 0.8%), 266 in the presumed EOS category (19.6%), and 252 in the control group, without sepsis (18.6%) (Figure 1). The incidence of presumed EOS in group A was 50.12%, while in group B was 50.28%.

A significant difference was found among the groups and each of the subgroups regarding the GA and birth weight (BW), as shown in Table 1 and Table 2.

Antibiotics were more frequently administered to neonates in the group with PROM ≥ 18 h (*p* < 0.001). Duration of hospital stay was significantly longer for patients in group A compared to group B (15.37 ± 21.54 days vs. 7.67 ± 12.98 days, *p* < 0.001).

The Apgar scores were lower in neonates with proven sepsis (Subgroups A1 and B1) compared to those with probable sepsis or without sepsis, irrespective of the duration of ruptured membranes (Table 2).

Moreover, a statistically significant difference was found when comparing the Apgar scores of group A with group B (*p* < 0.001). The proportion of preterm newborns was higher in the proven EOS group. Prolonged rupture of membranes ≥ 18 h was associated with longer hospital stays in all subgroups (Table 2).

Analysis of perinatal risk factors for EOS revealed that the number of positive amniotic fluid cultures was significantly higher in group A (*p* = 0.001) compared to group B. Subgroup analysis revealed that a positive amniotic fluid culture was associated only with probable EOS (*p* = 0.001), but no statistical significance was found between subgroups A1/B1 or A3/B3.

Furthermore, 12 of the mothers in subgroup A2 had chorioamnionitis, compared to none in the B2 subgroup (*p* = 0.005). Also, there were no statistically significant differences concerning other analyzed risk factors such as foul-smelling amniotic fluid, maternal fever, or maternal inflammatory syndrome between neonates in either subgroup (Table 3).

Regarding neonatal complications, respiratory distress syndrome (RDS) and retinopathy of prematurity (ROP) were significantly more frequent in group A than in group B (*p* < 0.001). Pneumothorax and pulmonary hemorrhage were statistically significant in subgroup A2 compared to group B2 (*p* = 0.002). Complications including mortality and patients included in the study are shown in Table 4 One neonate in subgroup A1 died due to EOS with *Staphylococcus capitis* (*p* = 0.28).

When comparing subgroup A1 with B1 more infants required mechanical ventilation (50% vs. 27%; *p* = 0.806), for a longer period (13.8 vs. 3.6 days; *p* = 0.322). However, no statistical significance was found. Inotropic support was necessary for three infants (30%) of subgroup A1, and two infants (18.2%) of subgroup B1 (*p* = 0.525).

Of the total 1355 patients, microorganisms were identified in the blood samples of 21 neonates (subgroup A1: 10 infants, and subgroup B1: 11 infants). The most common pathogen responsible for EOS was *Staphylococcus* spp. (n = 6; 28%). Among neonates with EOS and PROM ≥ 18 h, *Escherichia coli* was the most frequently detected pathogen (n = 5; 50%), whereas in group B, the most prevalent pathogen was *Klebsiella pneumoniae* (n = 4; 36.3%) (Table 5).

A comparison of hematological and serum parameters between groups is summarized in Table 6. Significant leukocytosis and high CRP values measured on day 1 were encountered in group B, with ROM < 18 h (*p* < 0.001).

Table 7 summarizes the descriptive statistics and *t*-tests for the main serum and hematological biomarkers with repeated measurements among subgroups with proven EOS. The procalcitonin serum levels were significantly higher in the A1 subgroup compared to the B1 subgroup (*p* < 0.05). However, other evaluated parameters did not significantly differ between subgroups.

Table 8 summarizes the descriptive statistics and *t*-tests for the main serum and hematological biomarkers with repeated measurements among subgroups with suspected EOS and without neonatal sepsis. Leukocytosis and CRP serum values recorded on the first day were significantly higher in the B2 subgroup compared to the A2 subgroup (*p* < 0.05). Also, when taking into consideration the subgroups without neonatal sepsis, our results showed that leukocytosis on day 1, CRP on day 3, and fibrinogen levels on day 2 were significantly higher in the B3 subgroup compared to the A3 subgroup (*p* < 0.05). The procalcitonin serum levels were significantly higher in the A2 subgroup compared to the B2 subgroup (*p* < 0.05).

**Figure 2:**
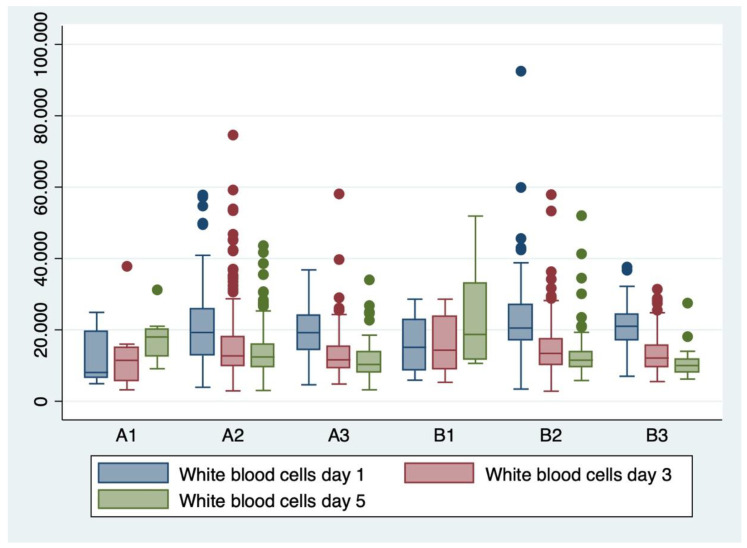
Boxplot representing the white blood cell count from days 1, 3, and 5 in the evaluated subgroups (A1–A3, B1–B3).

**Figure 3:**
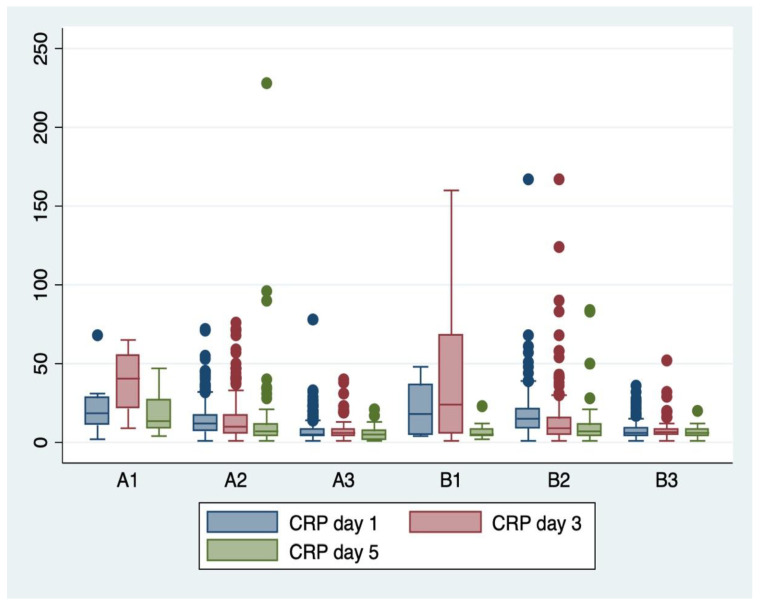
Boxplot representing the C-reactive protein (CRP) serum concentrations from days 1, 3, and 5 in the evaluated subgroups (A1–A3, B1–B3).

**Figure 4:**
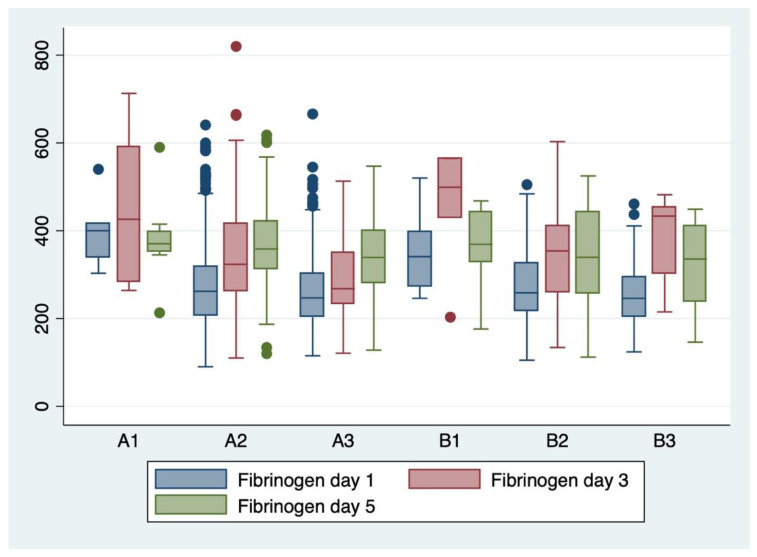
Boxplot representing the fibrinogen concentrations from days 1, 3, and 5 in the evaluated subgroups (A1–A3, B1–B3).

ANOVA analysis of variance along with the Bonferroni post-hoc multiple comparisons (Table 9) and boxplots (Figure 2, Figure 3 and Figure 4) were used for explanatory analysis of the differences in serum and hematological biomarkers concentrations (CRP, WBC, and fibrinogen) between subgroups. While ANOVA analysis revealed in the majority of cases a statistically significant difference in mean serum biomarker concentrations between at least two groups (*p* < 0.05). The Bonferroni post-hoc test found a statistically significant mean difference in white blood cell count from the first day between A2–B2 (*p* = 0.010; 95% CI: −16.24–−1.17), and A3–B3 subgroups (*p* = 0.022; 95% CI: −15.53–−0.64), serum CRP concentration from the first day between A2–B2 subgroups (*p* = 0.003; 95% CI: −5.47–−0.64), serum CRP concentration from the fifth day between A3-B3 subgroups (*p* = 0.04; 95% CI: 10.27–39.14), and serum fibrinogen concentration from the third day between A2–B2 subgroups (*p* = 0.008; 95% CI: 0.86–10.36), respectively.

A Random Effects GLS regression was used to measure the association between predictor variables, such as serum and hematological biomarkers levels (WBC, CRP, and fibrinogen) measured on three different occasions, and an outcome variable, proven early-onset neonatal sepsis (Table 10). Our model had a *p* value < 0.001, an R-squared value within groups of 0.47, and between groups of 0.82, while rho was 0.42. The statistically significant predictors of neonatal sepsis were the white blood cell count from the first (*p* = 0.005) and third day (*p* = 0.028), and CRP values from the first day (*p* = 0.004).

Table 11 summarizes the AUC for proven sepsis of the WBC, CRP, and fibrinogen levels measured on three different occasions, as well as for I/T and procalcitonin measured on one occasion. Moreover, it summarizes the AUC values corresponding to various combinations of these biomarkers. Our results showed that procalcitonin (AUC: 0.78) and CRP measured on the first day (AUC: 0.76) had the best predictive performance for early-onset neonatal sepsis when taken individually. Moreover, the best predictive performance for this type of sepsis was obtained by the combinations of biomarkers WBC, CRP, and fibrinogen recorded on the first day (AUC: 0.83), and WBC, CRP, and fibrinogen recorded on the third day (AUC: 0.90), respectively. The corresponding plots of AUC are presented as Supplementary Materials (Figures S1–S15).

## 4. Discussion

In order to prevent the morbidity and mortality related to EOS, identifying the main risk factors along with early diagnosis and therapy are crucial. Along with PROM, GA < 30 weeks, male sex, birth weight < 1500 g, inadequate prenatal care, low socio-economic status of the mother, poor maternal nutrition, maternal substance abuse, clinical chorioamnionitis, and lack of intrapartum antibiotics were cited as risk factors for neonatal sepsis [18,35].

Our study revealed that prematurity was significantly more frequent in the EOS group (*p* < 0.001). We found that out of 16 (76%) preterm neonates who developed EOS, 9 patients (90%) had PROM ≥ 18 h, and 7 patients (63.7%) had ROM < 18 h. Consistent with the data reported in the literature, the present study also showed that in the subgroup with proven sepsis male infants were predominant (subgroup A1: n = 6, 60%; subgroup B1: n = 8, 72%, EOS subgroups: n = 14, 66%), although this parameter did not present statistical significance [36,37,38,39]. Male neonates were reported to be at higher risk for EOS, according to the study on the largest population (n = 56,261, 50.8%) and the one extended over the longest period (18 years) [39,40]. However, there are studies whose results have identified an equal gender ratio or a predominance of females [41,42,43].

Marks et al. reviewed the literature over a period of 40 years in order to estimate the time until obtaining positive blood cultures in EOS [44]. In a total of 6188 blood cultures (5848 neonates), 250 positive cultures were identified, of which 146 were contaminants. The majority of blood cultures (54%) were positive for *Group B Streptococcus* (GBS). Moreover, in their study, 7 out of 8 (20%) neonates positive for *Escherichia coli* died from EOS. Recent studies identified a change in the distribution of organisms causing EOS, with a predominance of gram-negative rods, especially *Escherichia coli* [45]. National Institute of Child Health and Human Birth (NICHD), identified an increase from 3.2 to 5.09/1000 in *Escherichia coli* EOS and a decrease in GBS sepsis from 5.9 to 2.08/1000 on a cohort of VLBW infants [46].

Our results were consistent with these changes, *Escherichia coli* (n = 5/10; 50%) being the agent most isolated in group A, followed by *Staphylococcus* spp. (n = 3/10; 30%), *Klebsiella pneumoniae* (n = 1/10; 10%), and *Streptococcus* spp. (n = 1/10; 10%). In contradiction to group A, the pathogens inducing sepsis in group B were *Klebsiella pneumoniae* (n = 4/11; 36.3%), *Staphylococcus* spp. (n = 3/11; 27.2%), *Streptococcus* spp. (n = 3/11; 27.2%), and *Listeria monocytogenes* (n = 1/11; 9%). Sabry et al. identified *Klebsiella pneumoniae* as the leading cause of EOS in term infants [47]. In another study on term infants, EOS was most frequently caused by *Group B Streptococcus*, *Escherichia coli,* and *Enterococcus* spp. [48]. By comparison, the present study showed the following bacteria in term infants: group A (*Staphylococcus epidermidis:* n = 1, 10%), whereas in group B there were two neonates with *Klebsiella pneumoniae* (27%), one with *Staphylococcus epidermidis* (18%), and one was positive for both of them. The rate of gram-negative infection was higher in term than in preterm infants (60% vs. 45%). In contrast, the rate of gram-positive sepsis was higher in preterm than in term infants (76% vs. 24%). Overall, gram-positive organisms were isolated in 12 patients (57%), and gram-negative pathogens were identified in the probes of 9 infants (42%).

However, there are still numerous studies that reported *Group B Streptococcus* to be the most prevalent cause of EOS, especially in term infants [49,50]. These also include studies conducted in Southern Europe [51]. In a Greek study, this happened after the exclusion of Coagulase-negative staphylococci (CoNS) which were predominant (28.6%) [51].

Our region ranked first in the European colonization rate (6–32%), followed by Eastern Europe (19–29%) and Western Europe (11–21%) [52]. In contradiction, the literature cites Coagulase-negative staphylococci as a characteristic complication of late onset sepsis (LOS) [53]. Of the total of 21 infants with EOS, the one who died had a blood culture positive for *Staphylococcus capitis*. Interestingly, this pathogen has been isolated in the intensive care units of seventeen countries. Given the increased drug resistance, the unfavorable prognosis was not surprising.

Another finding of this study revealed high rates of presumed early onset sepsis (50.12% in group A and 50.28% in group B) compared to others cited in the literature [54]. This could be explained by the fact that the number of positive blood cultures was very low in the examined cohort, thus resulting in the inclusion of the majority of newborns into the “presumed sepsis” category. Other contributing factors for these high rates of presumed sepsis could be represented by the use of antibiotic therapy for some patients with prolonged rupture of membranes, a lack of pregnancy follow-up, and reduced detection rates of bacterial growth on conventional blood cultures. Previous studies have shown that a multiplex polymerase chain reaction protocol for finding neonatal sepsis would be more useful for making the diagnosis with smaller amounts of blood and less time than conventional blood cultures [55,56]. Thus, the inclusion of this type of assay in medical centers that evaluate a large number of patients with a suspicion of neonatal sepsis would reduce the use of antibiotics and the associated costs for the treatment of these patients.

As both CRP and PCT are influenced by gestational age and birth weight, it is also important to take into consideration the optimal time of determination [54]. It was demonstrated that term infants have higher CRP than preterm [55]. Eschborn et al. reviewed the kinetics of PCT and CRP, and concluded that in order to rule out EOS, it is more conclusive to determine CRP and PCT at 12 h of life rather than immediately after birth [56]. They also emphasized that serial determinations of these markers and their correlation with clinical findings were needed to support the decision of antibiotic therapy.

Neonatal complications (asphyxia, meconium aspiration, shock, and intraventricular hemorrhage) and maternal risk factors (prolonged labor, PROM) may also cause increase values of CRP [57]. In this context, there has been a constant quest for other markers that could be included in the laboratory panels used for the evaluation of newborns suspected of EOS. Newer studied markers like endocan, have elevated levels in newborns with sepsis (both early-onset and late-onset) and it is not influenced by gestational age, sex, fetal distress (meconium-stained amniotic fluid), delivery method, or minor birth trauma [58,59,60].

According to recent studies, presepsin may be a better marker than CRP and PCT for the diagnosis of EOS and for monitoring the response to therapy. Its value rises early in the umbilical cord blood of newborns with PPROM, does not vary with GA, postnatal age, or perinatal factors, and decreases progressively with the administration of antibiotics [61,62]. However, these biomarkers were not available for evaluation in our cohort of patients.

Another study that investigated WBC, PLT, and CRP in the diagnosis of EOS, reported normal values for WBC and PLT on both day 1 and day 3, but high values of CRP on both days, with an even elevated value on day 3 [41]. In our study, 7 (70%) patients with EOS (subgroup A1) showed high levels of CRP on days 1 and 3, while 6 (60%) of them had elevated CRP on day 5. The majority of neonates (n = 8; 72%) with EOS and PROM < 18 h showed high CRP levels on day 3. Furthermore, the results from our random Effects GLS regression indicated as statistically significant predictors of neonatal sepsis were the white blood cell count from the first (*p* = 0.005) and third day (*p* = 0.028), and CRP values from the first day (*p* = 0.004).

A biomarker is considered good if the AUC is higher than 0.75 or excellent if greater than 0.9, respectively. Hence, in our study, out of all the researched biomarkers, procalcitonin (AUC—0.78) and CRP measured on the first day (AUC—0.76) had the best predictive performance for early-onset neonatal sepsis. Even if we obtained good results, and procalcitonin is known as an early biomarker for neonatal sepsis, there are studies that state that it may also be increased in healthy newborns, and could be more accurate in diagnosing late-onset neonatal sepsis [63]. Moreover, our results showed that a combination of biomarkers (WBC, CRP, and fibrinogen), evaluated on the first and third day of life, had superior accuracy in detecting early onset neonatal sepsis (AUC: 0.83, AND 0.90, respectively).

Stocker et al. studied the relationship between the simultaneous determination of CRP, PCT, and WBC in no-sepsis, sepsis uncertain, sepsis probable, and sepsis-proven patients [43]. When comparing the proven sepsis group with the no sepsis one, they reported an AUC of 0.986 for CRP and an AUC of 0.921 for PCT, and those values increased with extended time frames up to 36 h, whereas there was no difference between start to 36 h vs. start to 48 h. In our study, although the AUC for CRP and PCT were lower, they did decrease between the first, second, and third determination. This change in the biomarker‘s dynamic could be the result of antibiotherapy.

This study has the following limitations: retrospective design, unbalanced data for the proven sepsis subgroups, and a limited number of biomarkers included. Further studies, on larger cohorts of neonates with early onset sepsis, that would include multiple panels of biomarkers could offer a more consistent perspective over the topic.

## 5. Conclusions

Neonatal sepsis is a condition that can pose important challenges to neonatologists, and its prompt identification is needed in order to offer the best possible care.

In the absence of positive blood culture, it is important to guide the therapeutic approach based on clinical manifestations and paraclinical investigations (WBC, CRP, PCT, imaging studies, etc.) determined in dynamics during the first days of life.

The results of this retrospective study outline the feasibility of using procalcitonin and CRP measured on the first day in order to increase the detection rate of early-onset neonatal sepsis, in the absence of positive blood cultures. This approach would justify the prompt initiation of antibiotic therapy.

Our results also indicated that a combination of biomarkers determined in the first and third day of life presented a superior predictive performance for early onset sepsis compared to markers evaluated independently, thus supporting the implementation of a combined panel of markers for the neonatal follow-up in case of sepsis suspicion.

## Figures and Tables

**Table 1:** Clinical and demographic characteristics of the study group.

Characteristics	PROM ≥ 18 h (Group A, n = 826)	ROM < 18 h (Group B, n = 529)	*p* Value
GA (weeks) mean ± SD	36.15 ± 3.9	37.83 ± 2.528	<0.001
Preterm (n/%) *	332 (40.19%)	86 (16.25%)	<0.001
BW (grams) mean ± SD	2730.3 ± 857.7	3146.3 ± 636.2	<0.001
CS (n/%)	371 (44.91%)	245 (46.31%)	0.615
VB (n/%)	455 (55.08%)	284 (53.68%)	0.614
Gender (n/%)	Male-419 (50.72%) Female-407 (49.27%)	Male-307 (58.03%) Female-222 (41.96%)	0.009
Apgar score, median and interquartile ranges	Apgar 1 min-7 (5–8) Apgar 5 min-8 (5–9) Apgar 10 min-8 (6–9)	Apgar 1 min-8 (6–9) Apgar 5 min-9 (7–10) Apgar 10 min-9 (8–10)	<0.001

Legend: PROM—prolonged rupture of membranes; ROM—rupture of membranes; GA—Gestational Age; BW—Birth Weight; CS—C-section; VB—vaginal birth; * GA < 37 weeks.

**Table 2:** Clinical and demographic characteristics of the newborns in the subgroups.

Characteristics	Proven EOS (A1) n = 10	Proven EOS (B1) n = 11	*p* Value	Presumed EOS (A2) n = 414	Presumed EOS (B2) n = 266	*p* Value	No Sepsis (A3) n = 402	No Sepsis (B3) n = 252	*p* Value
GA, WEEKS (mean ± SD)	29.5 ± 4.3	35.3 ± 3.1	0.002	35.6 ± 4.3	37.6 ± 3.1	<0.001	36.8 ± 3.1	38.1 ± 1.6	<0.001
Preterm * (n/%)	9 (90%)	7 (63.63%)	0.157	185 (44.68%)	46 (17.29%)	<0.001	138 (34.33%)	32 (12.70%)	<0.001
BW (median)	1484	2636	0.005	2605	3109	<0.001	2889	3250	<0.001
CS (n/%)	4 (40%)	8 (72.72%)	0.130	188 (45.41%)	123 (46.24%)	0.519	179 (44.53%)	114 (45.24%)	0.969
VB (n/%)	6 (60%)	3 (27.27%)	0.130	226 (54.59%)	143 (53.76%)	0.832	223 (55.47%)	138 (54.76%)	0.859
Gender (n/%)	Male-6 (60%) Female-4 (40%)	Male-8 (72.72%) Female-3 (27.27%)	0.014	204 (49.28%)	152 (57.14%)	0.045	213 (52.99%)	147 (58.33%)	0.181
				210 (50.72%)	114 (42.86%)	0.045	189 (47.01%)	105 (41.67%)	0.181
Apgar score, median and interquartile ranges	Apgar 1 min-6 (4–7) Apgar 5 min-7 (5–8) Apgar 10 min-7 (5–8)	Apgar 1 min-7 (5–8) Apgar 5 min-8 (7–9) Apgar 10 min-8 (7–9)	0.47	Apgar 1 min-8 (5–9) Apgar 5 min-8 (6–9) Apgar 10 min-8 (7–9)	Apgar 1 min-8 (7–9) Apgar 5 min-9 (8–10) Apgar 10 min-9 (8–10)	<0.001	Apgar 1 min-8 (7–9) Apgar 5 min-9 (7–10) Apgar 10 min-9 (7–10)	Apgar 1 min-9 (8–10) Apgar 5 min-9 (8–10) Apgar 10 min-9 (8–10)	<0.001
Duration of stay, days (mean ± SD)	52.10 ± 40.5	22.73 ± 12.1	<0.005	18.80 ± 24.6	9.75 ± 17.1	<0.001	10.92 ± 15.1	4.81 ± 3.8	<0.001

Legend: EOS—early onset sepsis; SD—standard deviation; GA—Gestational Age; BW—Birth Weight; CS—C-section; VB—vaginal birth; * GA < 37 weeks.

**Table 3:** Perinatal risk factors for infection.

Risk Factors	Proven EOS (A1) n = 10	Proven EOS (B1) n = 11	*p* Value	Presumed EOS (A2) n = 414	Presumed EOS (B2) n = 266	*p* Value	No Sepsis (A3) n = 402	No Sepsis (B3) n = 252	*p* Value
Positive amniotic fluid culture (n/%)	5 (50%)	0 (0%)	0.007	58 (14.01%)	15 (5.64%)	0.001	19 (4.73%)	8 (3.17%)	0.83
Foul smelling amniotic fluid (n/%)	2 (20%)	3 (27.27%)	0.28	41 (9.90%)	27 (10.15%)	0.41	16 (3.98%)	16 (6.35%)	0.739
Maternal fever (n/%)	1 (10%)	1 (9.09%)	0.94	6 (1.45%)	3 (1.13%)	0.72	1 (0.25%)	0 (0%)	0.428
Maternal inflammatory markers (n/%)	0 (0%)	0 (0%)	-	5 (1.21%)	6 (2.26%)	0.29	1 (0.25%)	0 (0%)	0.428

Legend: EOS—early onset sepsis.

**Table 4:** Neonatal mortality and complications in the studied subgroups.

Neonatal Complications	Proven EOS (A1) n = 10	Proven EOS (B1) n = 11	*p* Value	Presumed EOS (A2) n = 414	Presumed EOS (B2) n = 266	*p* Value	No Sepsis (A3) n = 402	No Sepsis (B3) n = 252	*p* Value
	Short term complications								
RDS (n/%)	9 (90%)	7 (63.63%)	0.157	134 (32.36%)	57 (21.42%)	0.002	68 (16.91%)	5 (1.98%)	<0.001
PPHN (n/%)	1 (10%)	0 (0%)	0.28	7 (1.69%)	7 (2.63%)	0.399	2 (0.49%)	0 (0%)	0.262
Pulmonary hemorrhage (n/%)	0 (0%)	1 (9.09%)	0.329	1 (0.24%)	1 (0.37%)	0.002	1 (0.24%)	0 (0%)	0.428
Pneumothorax (n/%)	1 (10%)	0 (0%)	0.366	8 (1.93%)	4 (1.50%)	0.002	2 (0.49%)	2 (0.79%)	0.636
Severe IVH * (n/%)	0 (0%)	1 (9.09%)	0.32	8 (1.93%)	6 (2.25%)	0.772	0 (0%)	0 (0%)	-
	Long term complications								
NEC (n/%)	1 (10%)	0 (0%)	0.28	4 (0.96%)	0 (0%)	0.049	0 (0%)	0 (0%)	0.428
ROP (n/%)	2 (20%)	0 (0%)	<0.001	24 (5.79%)	4 (1.50%)	0.006	4 (0.99%)	0 (0%)	0.112
BPD (n/%)	0 (0%)	1 (9.09%)	0.329	6 (1.44%)	4 (1.50%)	0.954	4 (0.99%)	0 (0%)	0.428
Antibiotherapy (days), mean ± SD	9.2 ± 4.36	10.7 ± 4.88	0.801	4.6 ± 1.83	8 ± 1.58	0.501	8 ± 1.63	5.8 ± 1.14	0.562
Duration of stay, (days), mean ± SD	52.10 ± 40.5	22.73 ± 12.1	<0.005	18.80 ± 24.6	9.75 ± 17.1	<0.001	10.92 ± 15.1	4.81 ± 3.8	<0.001
Mortality (n/%)	1 (10%)	0 (0%)	0.28	8 (1.93%)	7 (2.63%)	0.54	7 (1.74%)	0 (0%)	0.428

Legend: RDS—Respiratory distress syndrome; PPHN—persistent pulmonary hypertension of the newborn; IVH—intraventricular hemorrhage; NEC—necrotizing enterocolitis; ROP—retinopathy of prematurity; BPD—bronchopulmonary dysplasia; severe IVH * > grade III; EOS—early onset sepsis; SD—standard deviation.

**Table 5:** Comparison of blood culture results from the neonates with rupture of membranes.

Pathogen	PROM ≥ 18 h %	ROM < 18 h %	Total (%)
*Staphylococcus* spp.	3 (30%)	3 (27.3%)	6 (28.5%)
*Klebsiella pneumoniae*	1 (10%)	4 (36.4%)	5 (23.8%)
*Escherichia coli*	5 (50%)	0 (0%)	5 (23.8%)
*Streptococcus* spp.	1 (10%)	3 (27.3%)	4 (19.2%)
*Listeria monocytogenes*	0 (0%)	1 (9%)	1 (4.7%)

Legend: PROM—prolonged rupture of membranes, ROM—rupture of membranes.

**Table 6:** Comparison of the hematological and serum parameters between the groups.

Parameters	PROM > 18 h Group A			ROM < 18 h Group B			Independent *t*-Test	
	Mean ± SD			Mean ± SD			*t*	*p* Value
WBC × 10^3^/mm^3^ D1/D3/D5	19.54 ± 8.02	14.45 ± 8.21	13.1 ± 6.34	21.2 ± 7.87	14.34 ± 6.54	13.19 ± 7.61	−3.7 0.2 0.0	A vs. B D1: <0.001 A vs. B D3: >0.05 A vs. B D5: >0.05
I/T ratio	0.14 ± 0.11			0.15 ± 0.08			−0.53	A vs. B: >0.05
CRP mg/L D1/D3/D5	10.83 ± 9.56	11.86 ± 11.26	8.58 ± 15.06	13.02 ± 12.4	12.97 ± 18.35	8.51 ± 9.40	−3.3 −1.0 0.0	A vs. B D1: <0.001 A vs. B D3: >0.05 A vs. B D5: >0.05
Fibrinogen D1/D3/D5	270 ± 98.3	332 ± 118.6	362 ± 88.3	268 ± 79.8	362 ± 114.2	340 ± 108.9	0.16 −1.7 1.4	A vs. B D1: >0.05 A vs. B D3: >0.05 A vs. B D5: >0.05

Legend: PROM—prolonged rupture of membranes; ROM—rupture of membranes; WBC—white blood cells; CRP—c-reactive protein; I/T—immature/total neutrophils ratio; D—day; SD—standard deviation; vs.—versus.

**Table 7:** Comparison of the serum and hematological biomarkers measurements for the proven sepsis subgroups.

PROVEN SEPSIS Subgroup A1 Subgroup B1	Day 1	Day 3	Day 5	Independent *t*-Test	
	Mean ± SD	Mean ± SD	Mean ± SD	*t*	*p* Value
WBC × 10^3^/mm^3^	12.05 ± 7.74	13.15 ± 10.89	17.71 ± 6.98	−0.8 −0.5 −1.0	A1 vs. B1 D1: >0.05 A1 vs. B1 D3: >0.05 A1 vs. B1 D5: >0.05
	15.1 ± 8.03	15.49 ± 8.13	23.67 ± 15		
Procalcitonin (ng/mL)	17.32 ± 3.16			3.72	A1 vs. B1: <0.05
	12.78 ± 2.42				
I/T ratio	0.20 ± 0.08			2.0	A1 vs. B1: >0.05
	0.12 ± 0.04				
CRP mg/L	21.5 ± 19.10	38.5 ± 18.75	20.64 ± 14.25	0.4 0.0 2.1	A1 vs. B1 D1: >0.05 A1 vs. B1 D3: >0.05 A1 vs. B1 D5: >0.05
	19.57 ± 16.93	38.36 ± 7.56	47.88 ± 6.48		
Fibrinogen	399 ± 81.4	450 ± 191	380 ± 103	0.7 −0.0 0.3	A1 vs. B1 D1: >0.05 A1 vs. B1 D3: >0.05 A1 vs. B1 D5: >0.05
	355 ± 109.4	457 ± 125	358 ± 104		

Legend: WBC—white blood cells; CRP—c-reactive protein; I/T—immature/total neutrophils ratio; SD—standard deviation.

**Table 8:** Comparison of the serum and hematological biomarkers measurements for the subgroups with presumed sepsis or no sepsis.

Presumed EOS Subgroup A2 Subgroup B2	Day 1	Day 3	Day 5	Independent *t*-Test	
	Mean ± SD	Mean ± SD	Mean ± SD	*t*	*p* Value
WBC × 10^3^/mm^3^	20.13 ± 9.15	13.92 ± 9.15	15.31 ± 9.05	−2.4 0.6 0.7	A2 vs. B2 D1: <0.05 A2 vs. B2 D3: >0.05 A2 vs. B2 D5: >0.05
	21.9 ± 9.18	13.23 ± 6.9	14.83 ± 7.09		
Procalcitonin (ng/mL)	9.12 ± 1.26			4.84	A2 vs. B2: < 0.05
	4.37 ± 1.14				
I/T ratio	0.14 ± 0.12			−0.4	A2 vs. B2: >0.05
	0.15 ± 0.09				
CRP (mg/L)	13.96 ± 10.17	13.4 ± 6.7	17.6 ± 9.78	−3.0 −0.3 0.2	A2 vs. B2 D1: <0.05 A2 vs. B2 D3: >0.05 A2 vs. B2 D5: >0.05
	17 ± 4.37	13.8 ± 9.32	18 ± 10.7		
Fibrinogen	277 ± 104	343 ± 120	364 ± 87.6	0.1 −0.0 1.3	A2 vs. B2 D1: >0.05 A2 vs. B2 D3: >0.05 A2 vs. B2 D5: >0.05
	275 ± 84.7	344 ± 109	342 ± 110		
**No sepsis** **Subgroup A3** **Subgroup B3**	**Day 1** **Mean ± SD**	**Day 3** **Mean ± SD**	**Day 5** **Mean ± SD**	***t***	***p* value**
WBC × 10^3^/mm^3^	19.09 ± 6.44	13.02 ± 6.13	11.42 ± 4.80	−3.0 −0.5 0.9	A3 vs. B3 D1: <0.05 A3 vs. B3 D3: >0.05 A3 vs. B3 D5: >0.05
	20.76 ± 5.57	13.39 ± 5.19	10.55 ± 3.99		
I/T ratio	0.104 ± 0.09			−3.5	A3 vs. B3: >0.05
	0.14				
CRP mg/L	7.03 ± 6.5	7.18 ± 5.77	4.99 ± 3.38	−0.9 −0.7 −2.0	A3 vs. B3 D1: >0.05 A3 vs. B3 D3: >0.05 A3 vs. B3 D5: <0.05
	7.57 ± 5.6	7.80 ± 6.93	6.22 ± 3.42		
Fibrinogen	257 ± 86	293 ± 92.67	350 ± 88	0.1 −2.5 0.8	A3 vs. B3 D1: >0.05 A3 vs. B3 D3: <0.05 A3 vs. B3 D5: >0.05
	255 ± 68.11	383 ± 99.59	320 ± 115		

Legend: EOS—early onset sepsis; WBC—white blood cells; CRP—c-reactive protein; I/T—immature/total neutrophils ratio; SD—standard deviation; vs.—versus.

**Table 9:** ANOVA analysis of variance and Bonferroni post-hoc test for the serum and hematological biomarkers for neonatal sepsis.

Serum Biomarkers	Groups	ANOVA Results		Bonferroni Test				
		F Score	*p* Value	Mean Difference	Standard Error	*p* Value	95% Confidence Interval Lower Limit	95% Confidence Interval Upper Limit
**WBC day 1**	A1–B1	7.13	<0.001	−3.05	3.45	1.000	−13.21	7.11
	A2–B2			−8.71	2.56	0.010	−16.24	−1.17
	A3–B3			−8.08	2.53	0.022	−15.53	−0.64
**WBC day 3**	A1–B1	2.79	0.016	−2.34	3.59	1.000	−12.92	8.24
	A2–B2			0.48	0.68	1.000	−1.53	2.50
	A3–B3			−0.037	0.90	1.000	−3.03	2.28
**WBC day 5**	A1–B1	8.27	<0.001	−5.96	3.23	0.98	−15.49	3.57
	A2–B2			0.68	0.82	1.000	−1.73	3.10
	A3–B3			0.87	1.28	1.000	−2.91	4.66
**CRP day 1**	A1–B1	41.43	<0.001	1.92	4.92	1.000	−12.55	16.40
	A2–B2			−3.05	0.82	0.003	−5.47	−0.64
	A3–B3			−0.54	0.91	1.000	−3.22	2.13
**CRP day 3**	A1–B1	21.4	<0.001	0.13	6.33	1.000	−18.50	18.78
	A2–B2			−0.39	1.16	1.000	−3.84	3.05
	A3–B3			−0.61	1.79	1.000	−5.91	4.68
**CRP day 5**	A1–B1	3.2	0.007	11.19	6.37	1.000	−7.59	29.98
	A2–B2			0.45	1.41	1.000	−3.72	4.62
	A3–B3			24.70	4.90	0.04	10.27	39.14
**Fibrinogen day 1**	A1–B1	5.2	<0.001	43.9	55.08	1.000	−118.29	206.09
	A2–B2			1.41	9.02	1.000	−25.16	27.98
	A3–B3			1.34	10.40	1.000	−29.28	31.97
**Fibrinogen day 3**	A1–B1	4.96	<0.001	−7.19	63.63	1.000	−195.52	181.14
	A2–B2			5.60	1.61	0.008	0.86	10.36
	A3–B3			−90.39	42.92	0.541	−217.45	36.65
**Fibrinogen day 5**	A1–B1	0.73	0.59	21.16	50.95	1.000	−130.06	172.39
	A2–B2			22.34	16.88	1.000	−27.75	72.44
	A3–B3			−1.23	2.34	1.000	−8.14	5.68

Legend: WBC—white blood cells; CRP—c-reactive protein; I/T—immature/total neutrophils ratio.

**Table 10:** Random Effects Generalized Least Squares regression of biochemical predictors for proven neonatal sepsis.

Serum Biomarkers	Coefficient	*p* Value	95% Confidence Interval
**WBC day 1**	0.98	0.005	0.016–0.09
**WBC day 3**	1.74	0.028	−0.08–−0.004
**WBC day 5**	−0.32	0.769	−0.04–0.03
**CRP day 1**	1.67	0.004	−0.01–0.02
**CRP day 3**	−0.19	0.063	−0.0008–0.03
**CRP day 5**	−0.46	0.483	−0.044–0.021
**Fibrinogen day 1**	−0.88	0.642	−0.002–0.001
**Fibrinogen day 3**	−0.15	0.823	−0.002–0.002
**Fibrinogen day 5**	−0.57	0.117	−0.004–0.0004

Legend: WBC—white blood cells; CRP—C-reactive protein.

**Table 11:** AUC for proven sepsis.

Biomarker	AUC Value	95% Confidence Interval
**WBC day 1**	0.55	0.35–0.76
**WBC day 3**	0.47	0.32–0.69
**WBC day 5**	0.49	0.36–0.68
**CRP day 1**	0.76	0.58–0.88
**CRP day 3**	0.66	0.47–0.85
**CRP day 5**	0.62	0.39–0.86
**Fibrinogen day 1**	0.34	0.28–0.55
**Fibrinogen day 3**	0.37	0.36–0.61
**Fibrinogen day 5**	0.35	0.24–0.63
**I/T**	0.58	0.41–0.74
**PCT**	0.78	0.69–0.93
**WBC + CRP + Fibrinogen (day 1)**	0.83	0.71–0.96
**WBC + CRP + Fibrinogen (day 3)**	0.90	0.84–0.95
**WBC + CRP + Fibrinogen (day 5)**	0.70	0.59–0.82
**I/T + PCT**	0.76	0.61–0.92

Legend: WBC—white blood cells; CRP—c-reactive protein; I/T—immature/total neutrophils ratio; PCT—procalcitonin; WBC + CRP + Fibrinogen—combination of serum biomarkers in day 1, 3 or 5.

## Data Availability

The data presented in this study are available on request from the corresponding author. The data are not publicly available due to local policies.

## Supplementary Materials

The following supporting information can be downloaded at: https://www.mdpi.com/article/10.3390/children11010124/s1, Figure S1. AUC WBC day 1; Figure S2. AUC WBC day 3; Figure S3. AUC WBC day 5; Figure S4. AUC CRP day 1; Figure S5. AUC CRP day 3; Figure S6. AUC CRP day 5; Figure S7. AUC FIB day 1; Figure S8. AUC FIB day 3; Figure S9. AUC FIB day 5; Figure S10. AUC I/T; Figure S11. AUC PCT; Figure S12. AUC I/T+PCT; Figure S13. AUC parameters day 1; Figure S14. AUC parameters day 3; Figure S15. AUC parameters day 5.
