# Supplementary figures and images for: Investigating the Association between Serum and Hematological Biomarkers and Neonatal Sepsis in Newborns with Premature Rupture of Membranes: A Retrospective Study
Source: Children (Basel). 2024 Jan 18;11(1):124. doi: 10.3390/children11010124 (PMC10814729; doi:10.3390/children11010124)

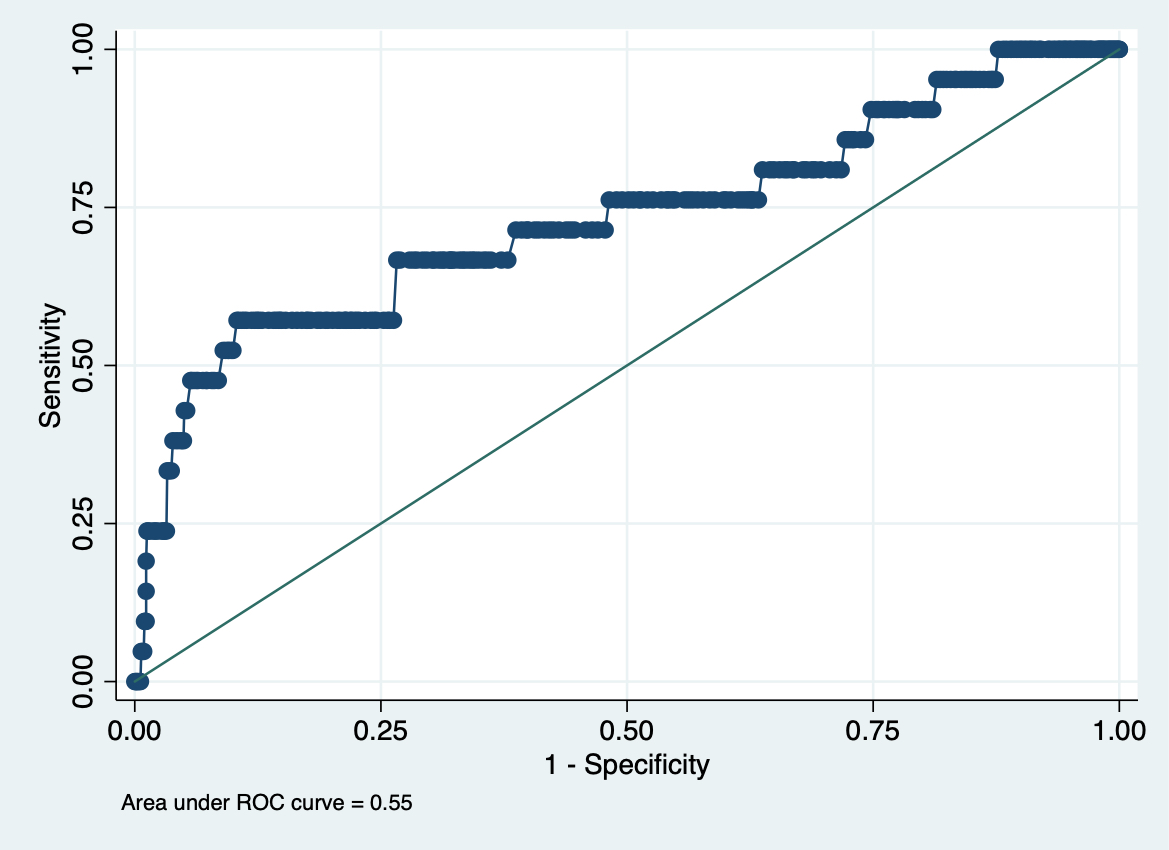

Supplement: Supplementary file 1 [file children-11-00124-s001.zip › children-neonat-supplementary/S1_AUC WBC day 1.jpg]

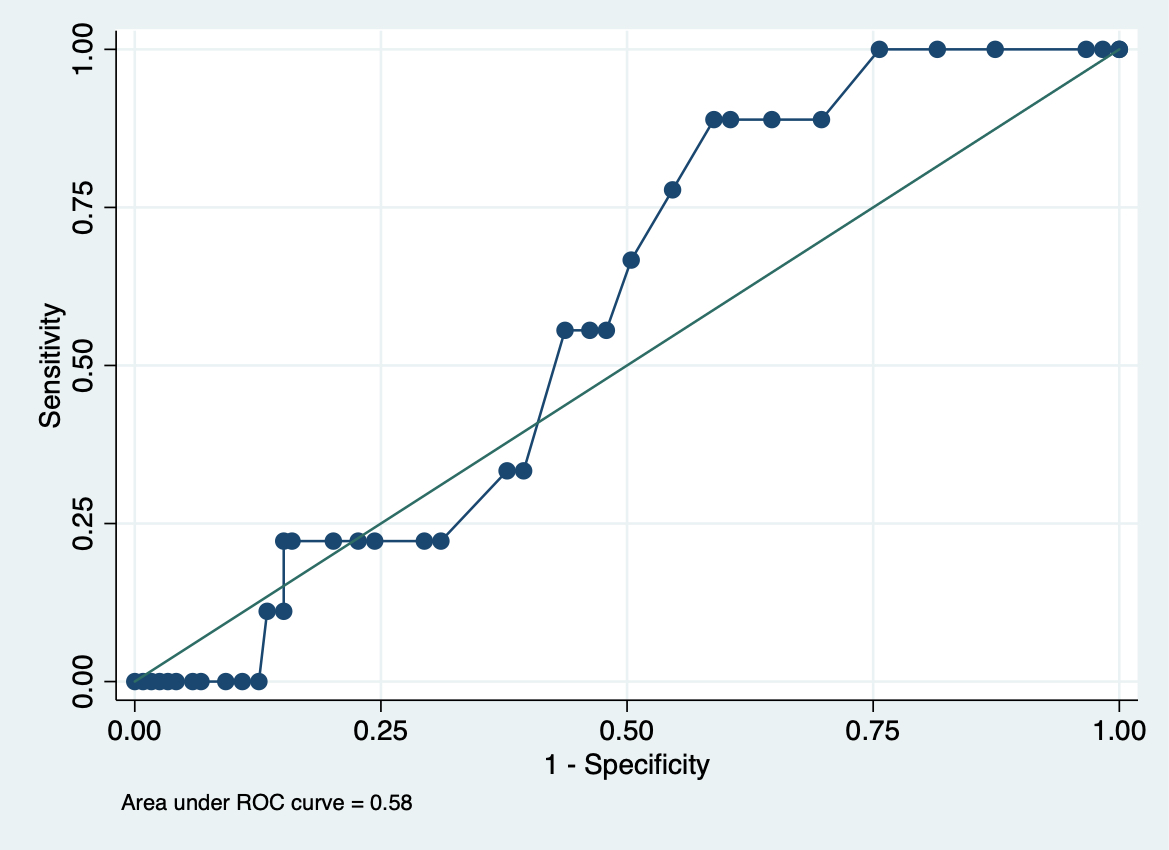

Supplement: Supplementary file 1 [file children-11-00124-s001.zip › children-neonat-supplementary/S10_AUC I_T.jpg]

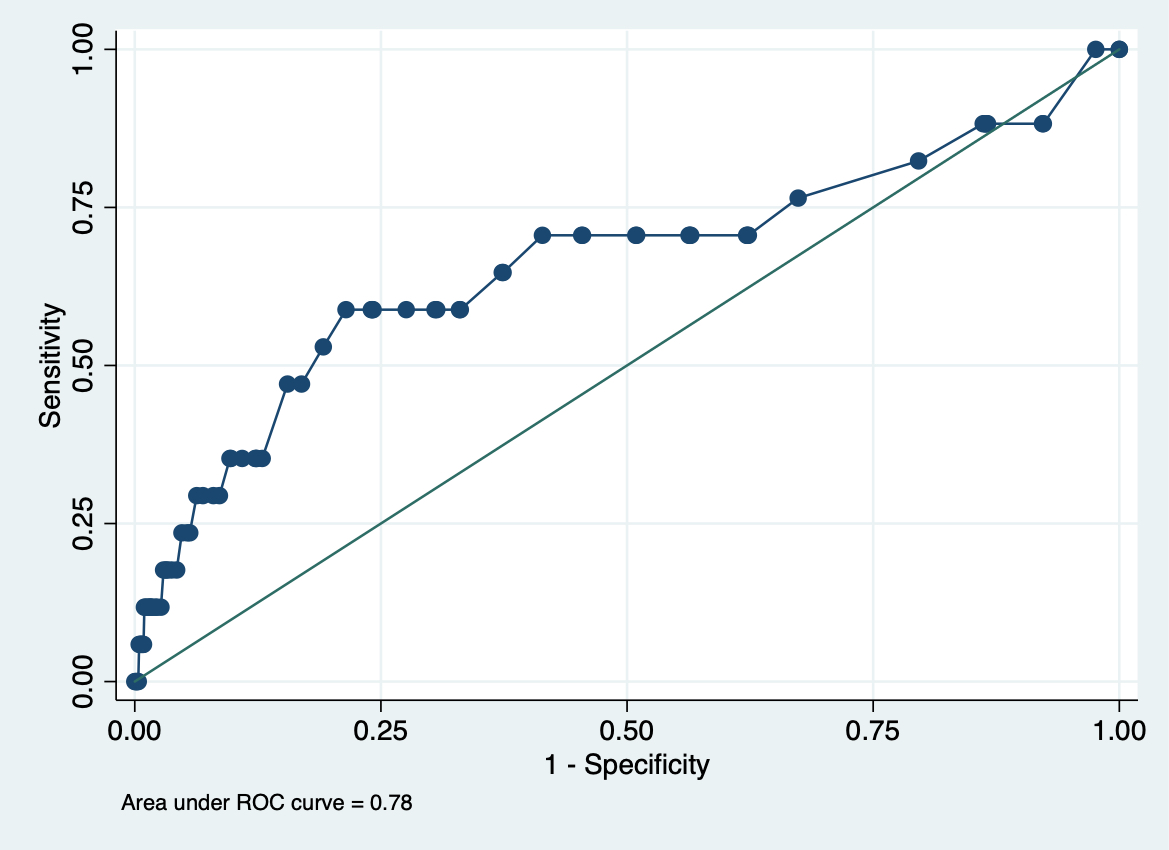

Supplement: Supplementary file 1 [file children-11-00124-s001.zip › children-neonat-supplementary/S11_AUC PCT.jpg]

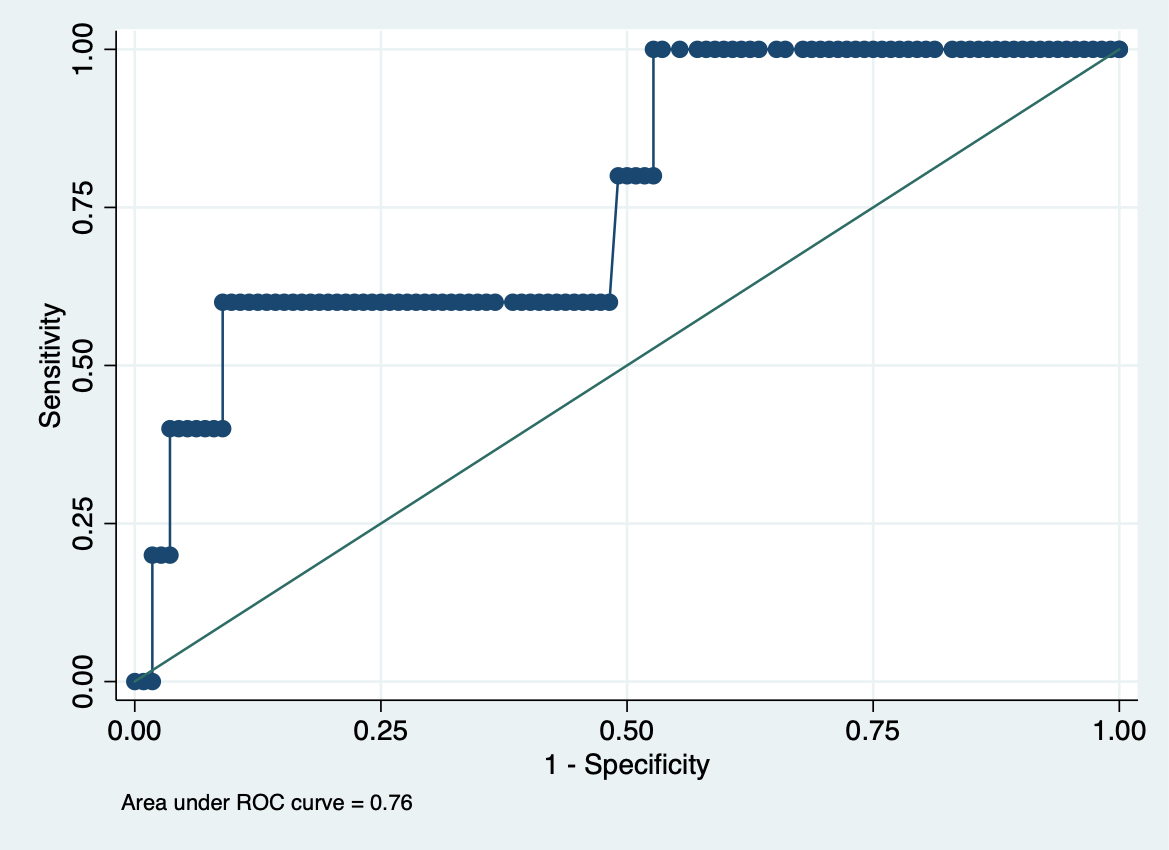

Supplement: Supplementary file 1 [file children-11-00124-s001.zip › children-neonat-supplementary/S12_AUC I_T+PCT.jpg]

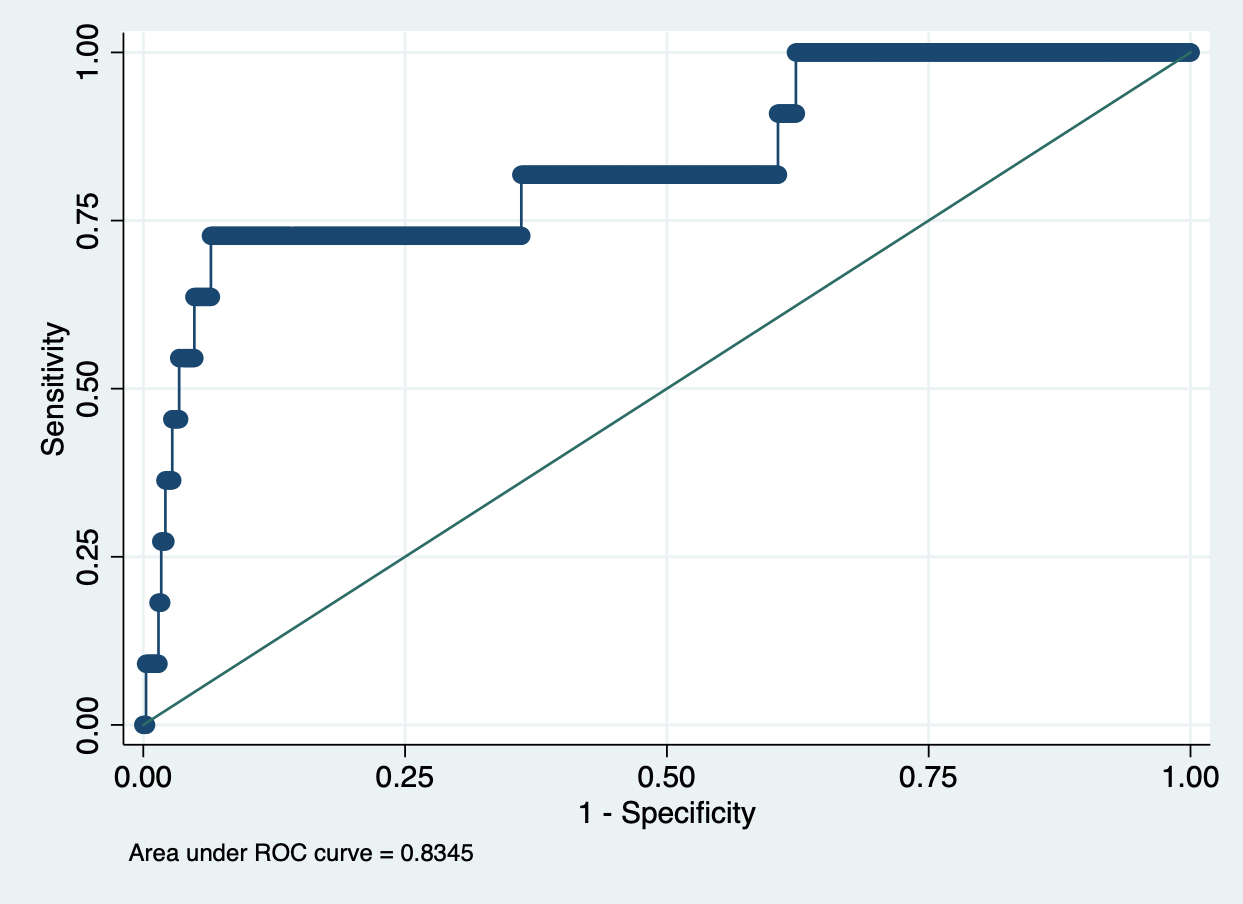

Supplement: Supplementary file 1 [file children-11-00124-s001.zip › children-neonat-supplementary/S13_AUC parametres day 1.jpg]

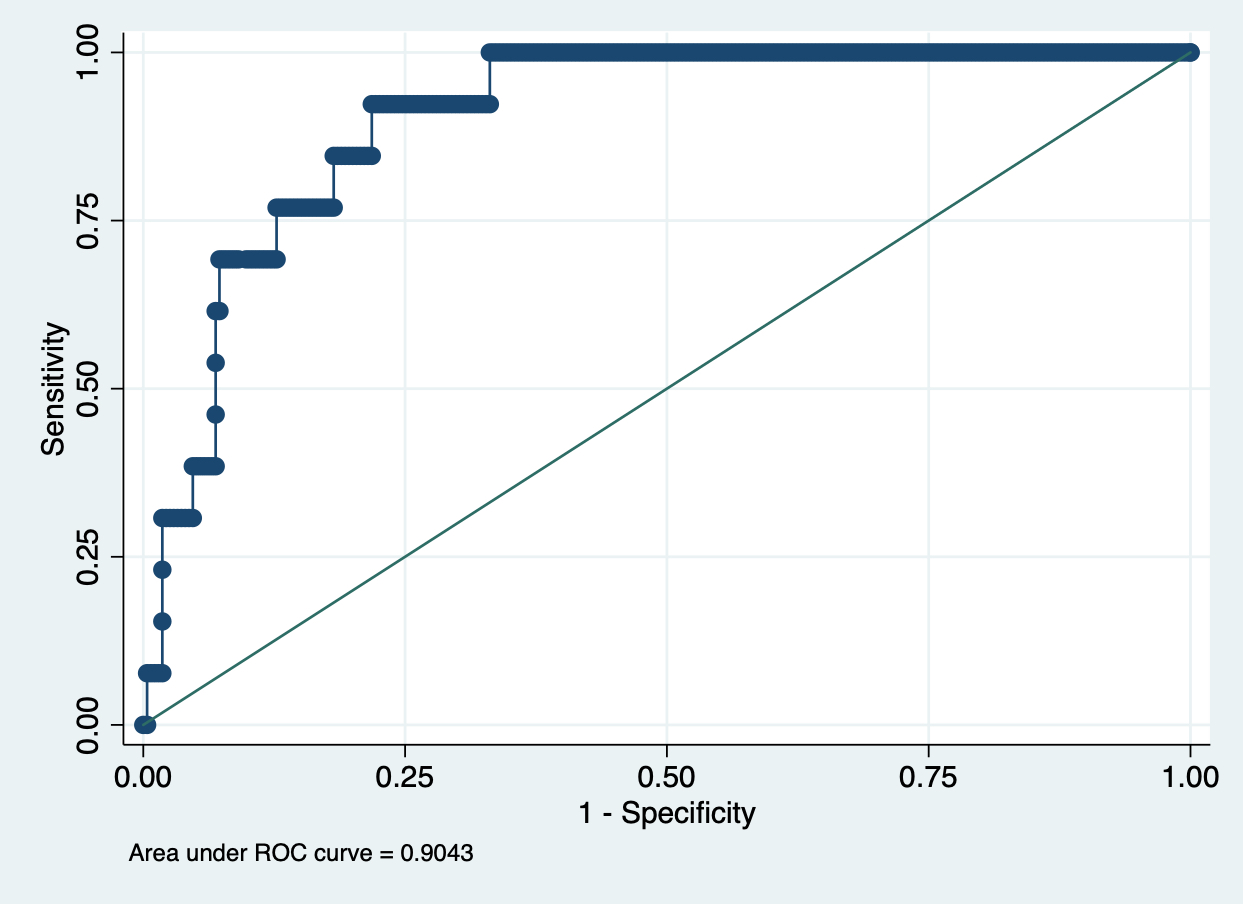

Supplement: Supplementary file 1 [file children-11-00124-s001.zip › children-neonat-supplementary/S14_AUC parametres day 3.jpg]

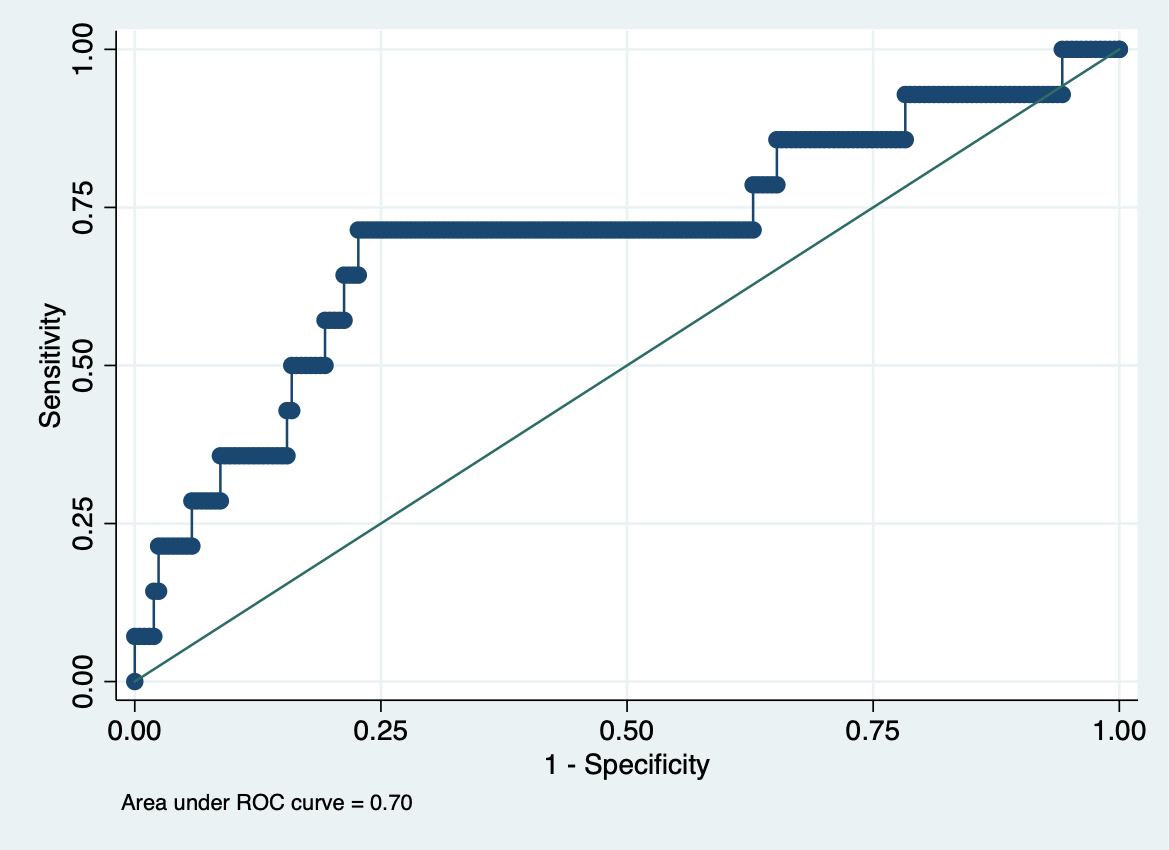

Supplement: Supplementary file 1 [file children-11-00124-s001.zip › children-neonat-supplementary/S15_AUC parametres day 5.jpg]

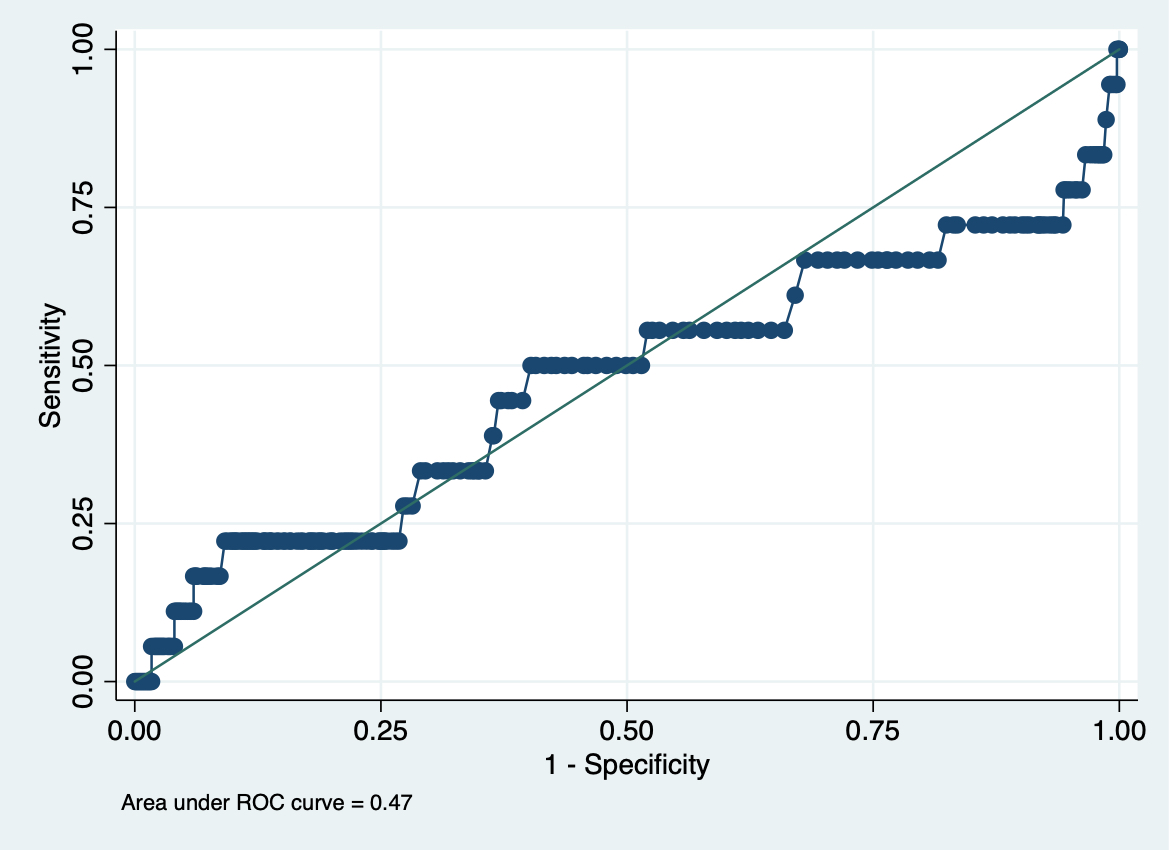

Supplement: Supplementary file 1 [file children-11-00124-s001.zip › children-neonat-supplementary/S2_AUC WBC day 3.jpg]

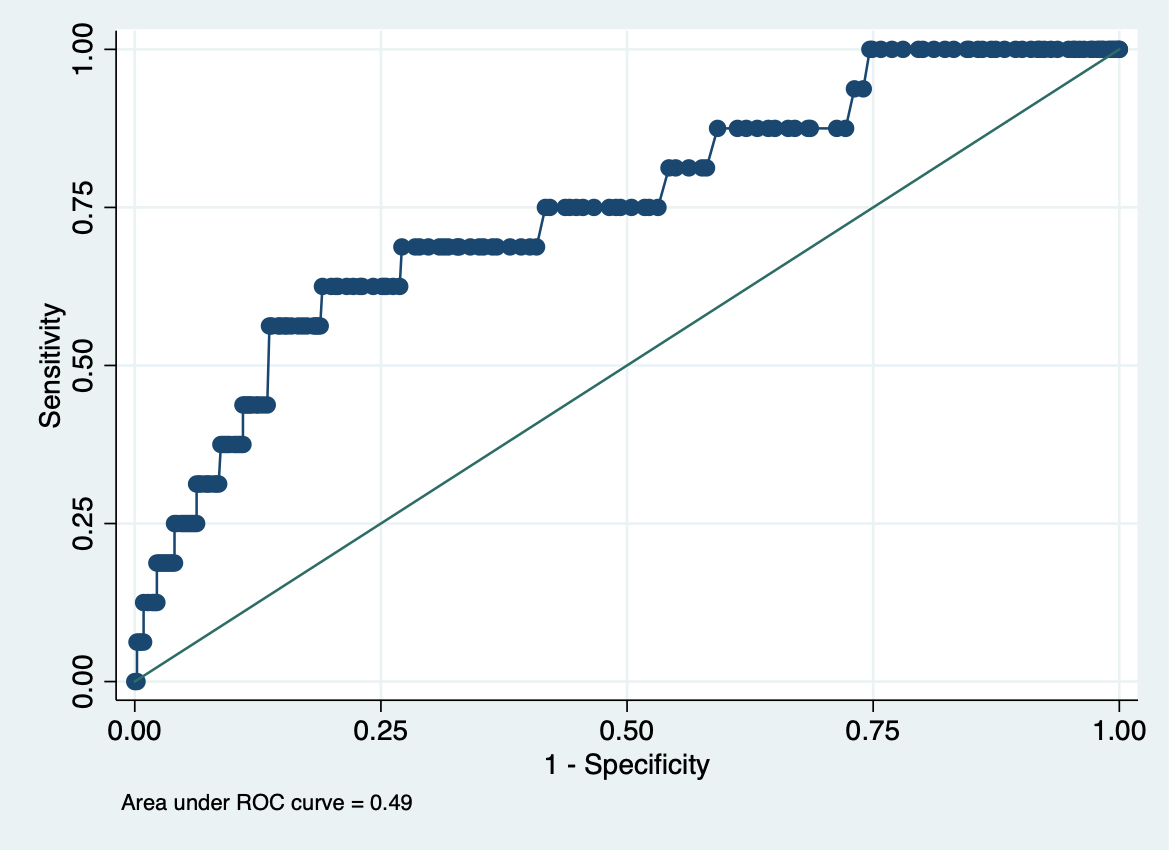

Supplement: Supplementary file 1 [file children-11-00124-s001.zip › children-neonat-supplementary/S3_AUC WBC day 5.jpg]

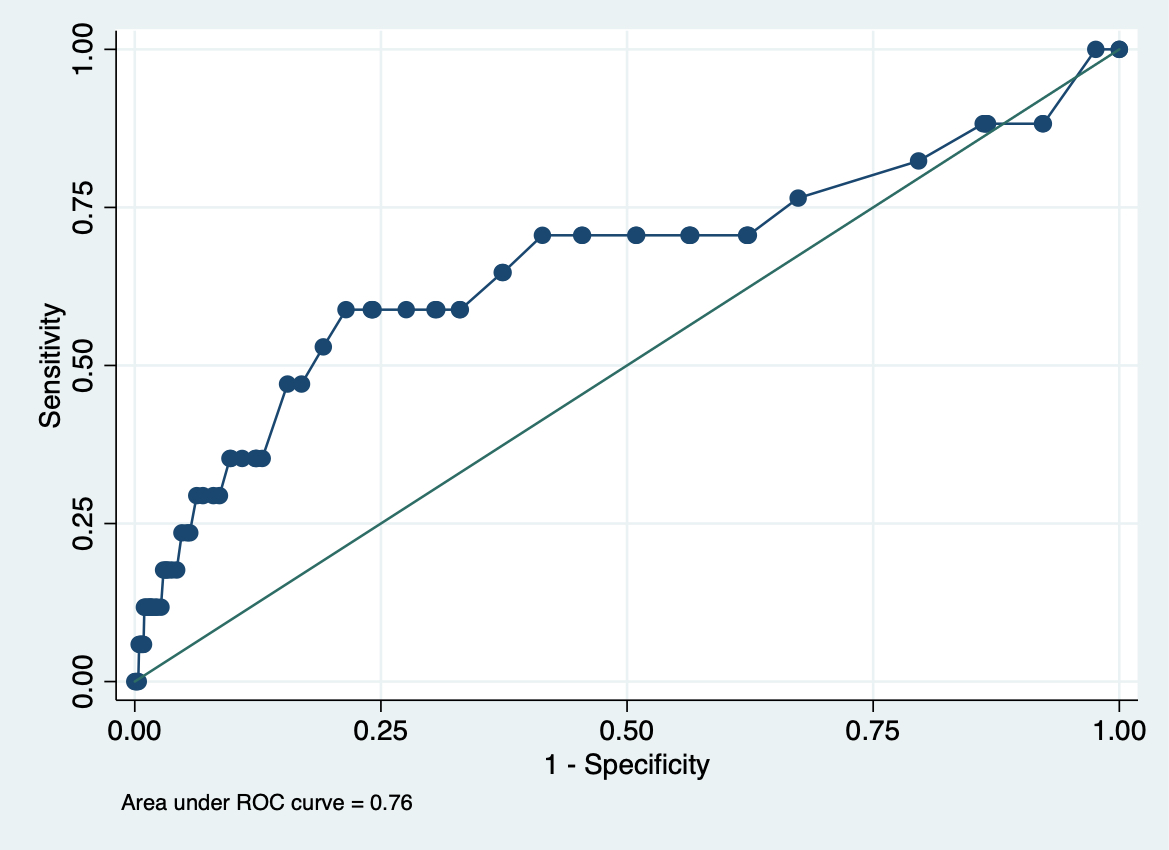

Supplement: Supplementary file 1 [file children-11-00124-s001.zip › children-neonat-supplementary/S4_AUC CRP day 1.jpg]

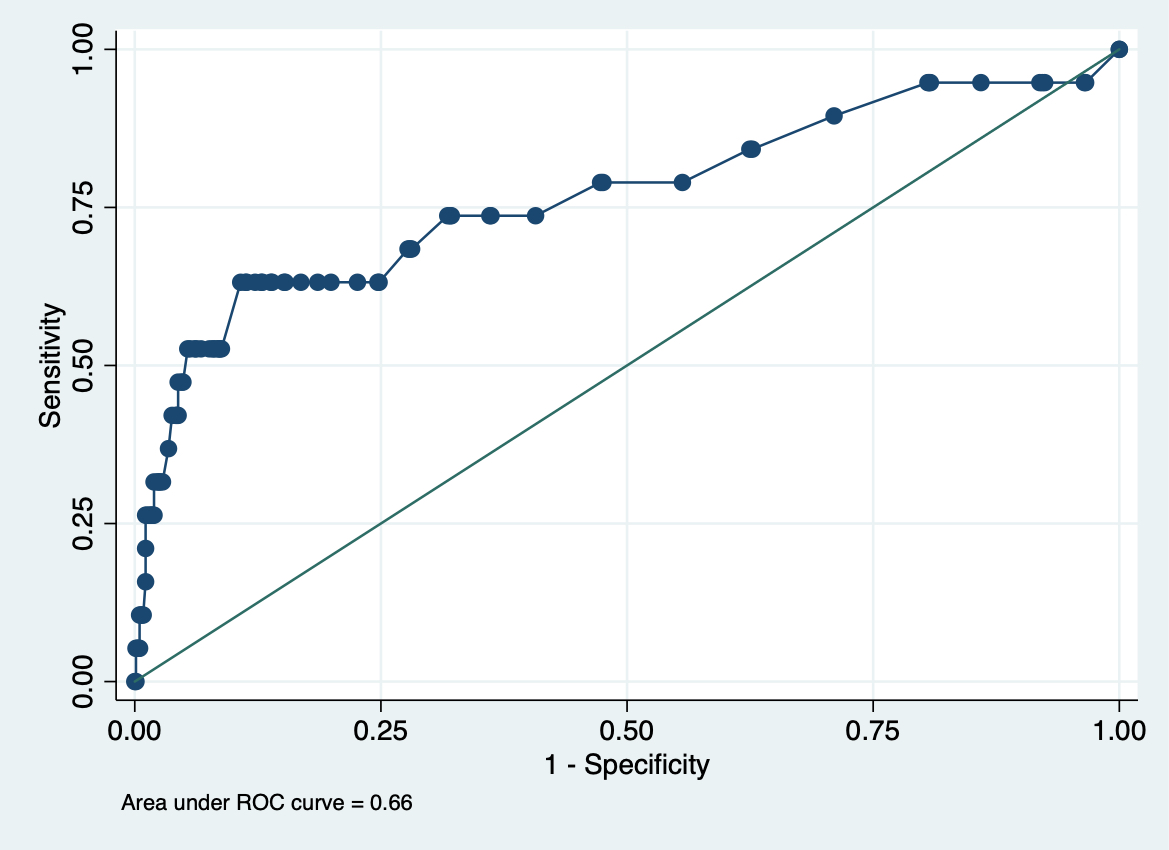

Supplement: Supplementary file 1 [file children-11-00124-s001.zip › children-neonat-supplementary/S5_AUC CRP day 3.jpg]

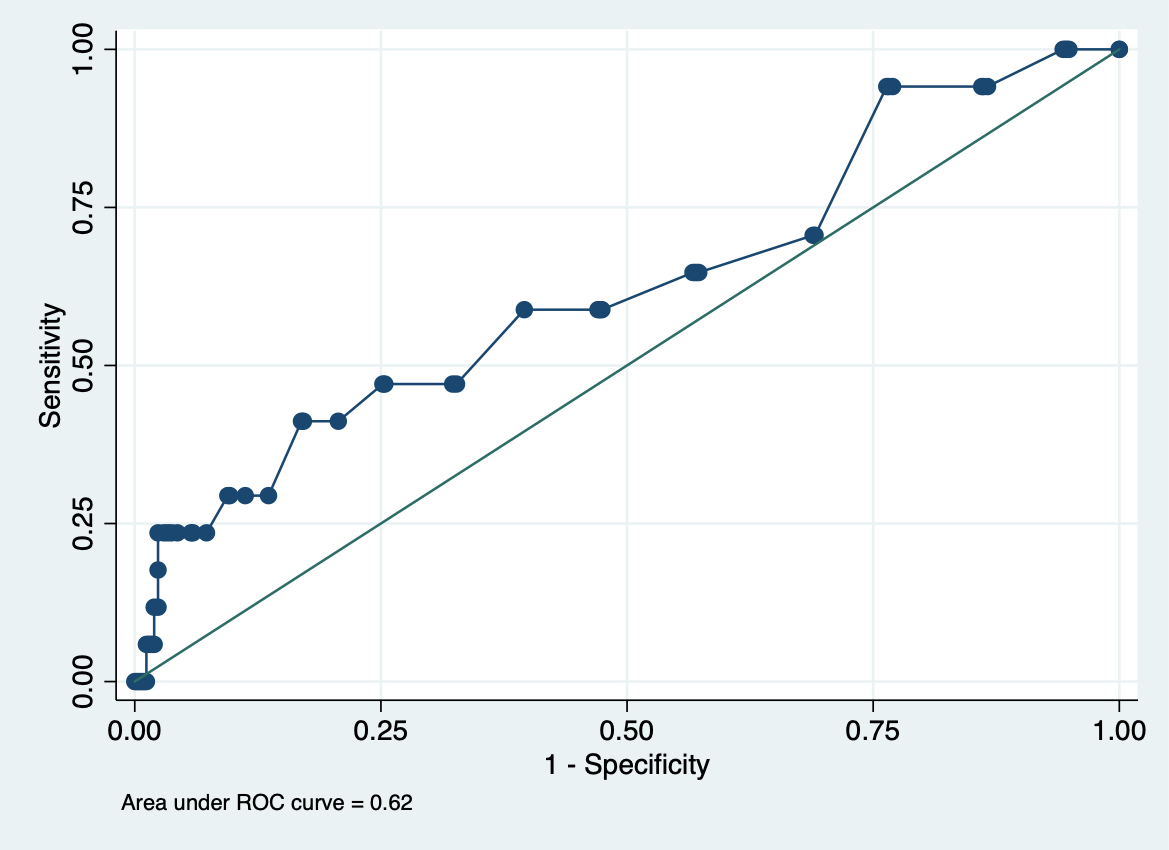

Supplement: Supplementary file 1 [file children-11-00124-s001.zip › children-neonat-supplementary/S6_AUC CRP day 5.jpg]

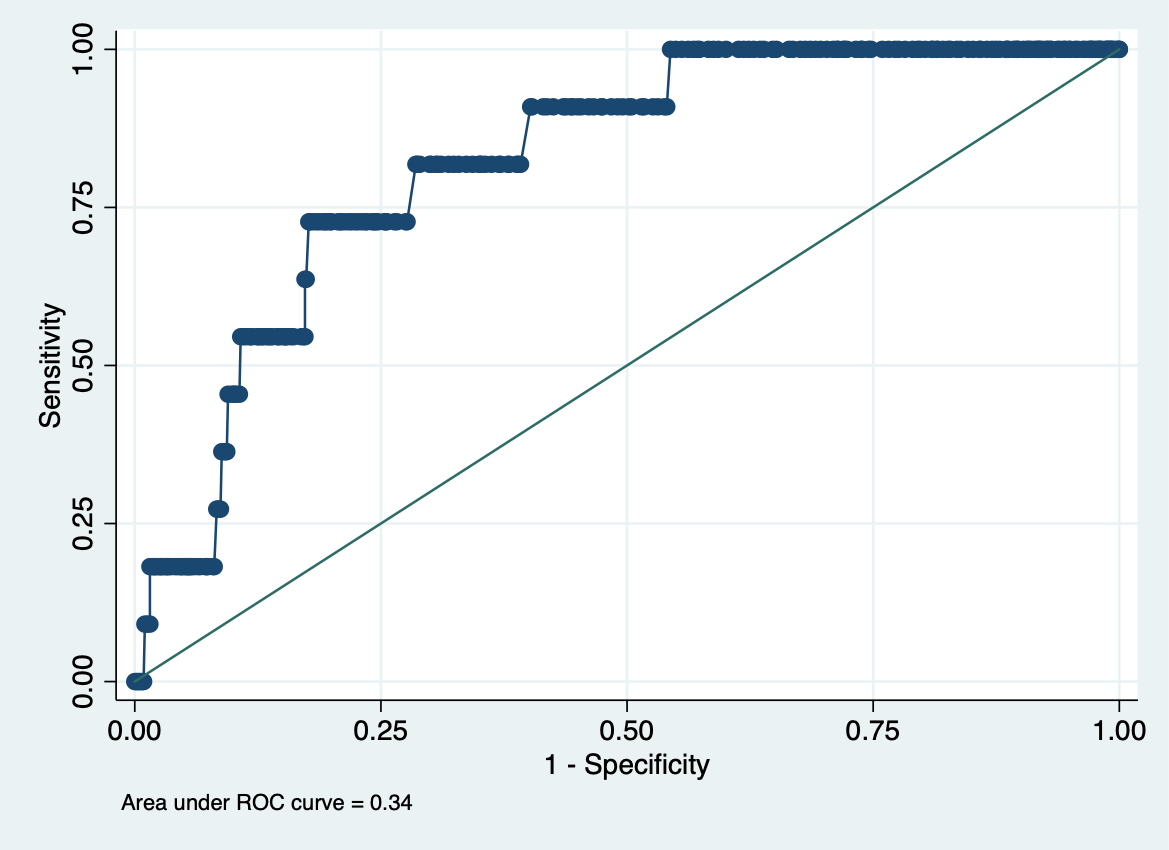

Supplement: Supplementary file 1 [file children-11-00124-s001.zip › children-neonat-supplementary/S7_AUC FIB day 1.jpg]

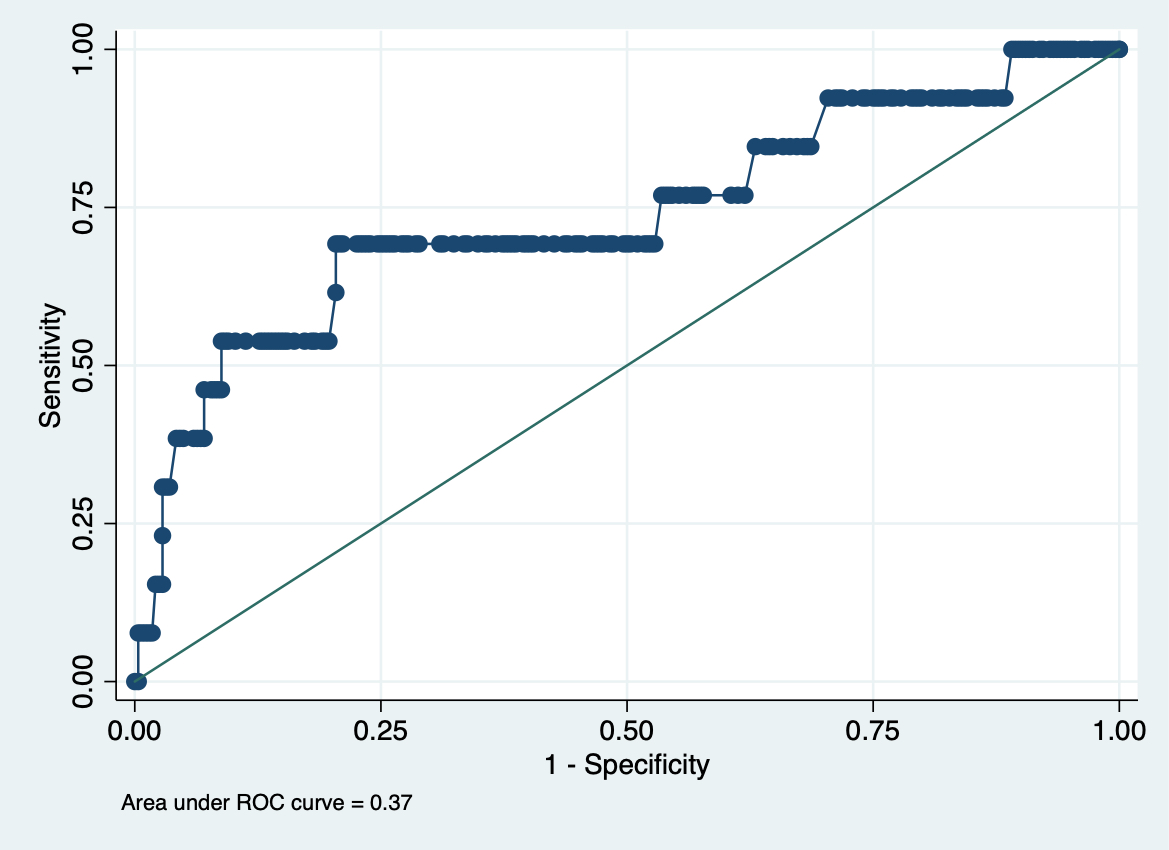

Supplement: Supplementary file 1 [file children-11-00124-s001.zip › children-neonat-supplementary/S8_AUC FIB day 3.jpg]

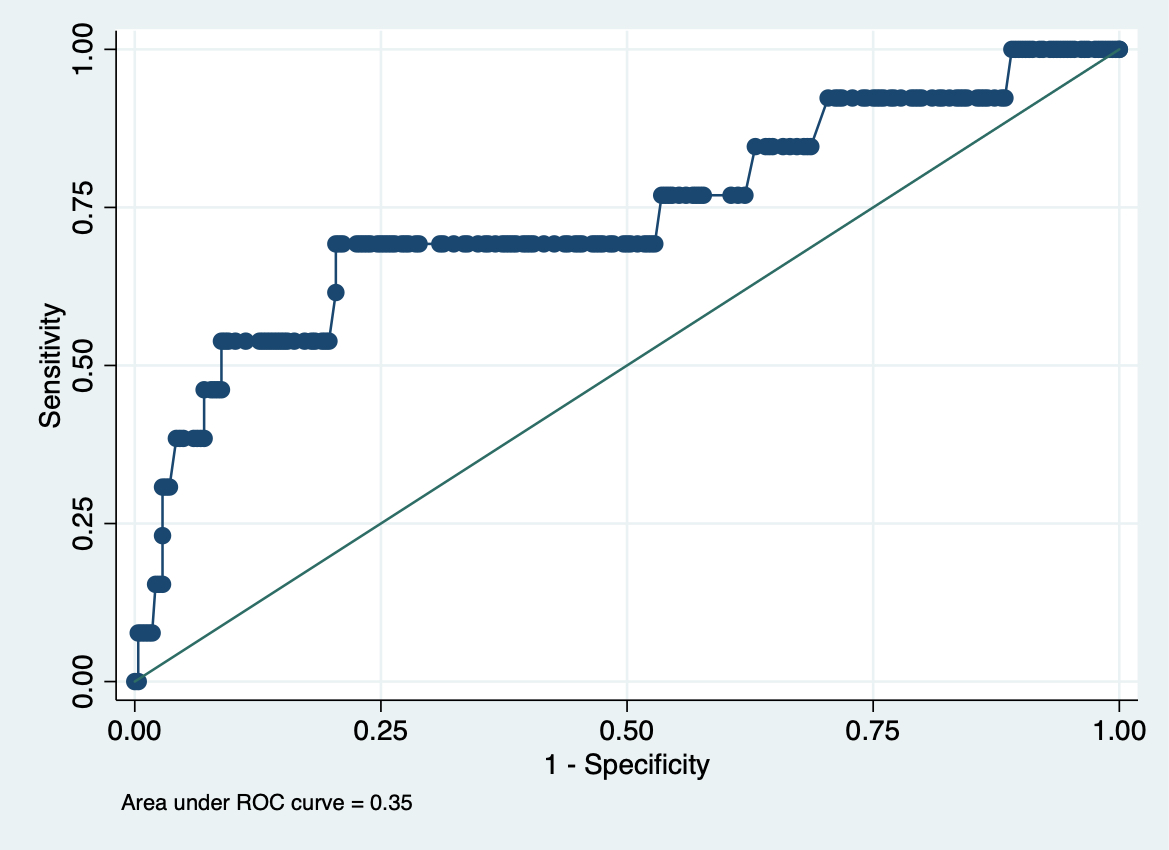

Supplement: Supplementary file 1 [file children-11-00124-s001.zip › children-neonat-supplementary/S9_AUC FIB day 5.jpg]
